# Printability Study of a Conductive Polyaniline/Acrylic Formulation for 3D Printing

**DOI:** 10.3390/polym13132068

**Published:** 2021-06-23

**Authors:** Goretti Arias-Ferreiro, Ana Ares-Pernas, Aurora Lasagabáster-Latorre, Nora Aranburu, Gonzalo Guerrica-Echevarria, M. Sonia Dopico-García, María-José Abad

**Affiliations:** 1 Grupo de Polímeros, Centro de Investigacións Tecnolóxicas, Universidade da Coruña, Campus de Ferrol, 15471 Ferrol, Spain; goretti.arias@udc.es (G.A.-F.); ana.ares@udc.es (A.A.-P.); s.dopico@udc.es (M.S.D.-G.); 2 Departemento Química Orgánica I, Facultad de Óptica y Optometría, Universidad Complutense de Madrid, Arcos de Jalón 118, 28037 Madrid, Spain; aurora@ucm.es; 3 POLYMAT and Department of Advanced Polymers and Materials, Physics, Chemistry and Technology, Faculty of Chemistry, University of the Basque Country UPV/EHU, 20018 San Sebastián, Spain; nora.aramburu@ehu.eus (N.A.); gonzalo.gerrika@ehu.eus (G.G.-E.)

**Keywords:** polyaniline, UV curing, acrylic conductive composite, 3D printing, vat polymerization, DLP

## Abstract

There is need for developing novel conductive polymers for Digital Light Processing (DLP) 3D printing. In this work, photorheology, in combination with Jacobs working curves, efficaciously predict the printability of polyaniline (PANI)/acrylate formulations with different contents of PANI and photoinitiator. The adjustment of the layer thickness according to cure depth values (*C_d_*) allows printing of most formulations, except those with the highest gel point times determined by photorheology. In the working conditions, the maximum amount of PANI embedded within the resin was ≃3 wt% with a conductivity of 10^−5^ S cm^−1^, three orders of magnitude higher than the pure resin. Higher PANI loadings hinder printing quality without improving electrical conductivity. The optimal photoinitiator concentration was found between 6 and 7 wt%. The mechanical properties of the acrylic matrix are maintained in the composites, confirming the viability of these simple, low-cost, conductive composites for applications in flexible electronic devices.

## 1. Introduction

Additive manufacturing (AM) or 3D printing technologies are gaining importance in the industry due to the new production models that establish the paradigms of Industry 4.0. However, one of the major drawbacks of AM remains—its limited materials selection portfolio, with low availability of high-performance materials with specific properties. Therefore, more research is required on providing materials with new features [1,2,3] with the minimum environmental cost, as demanded by today’s society. In this way, the use of 3D printing technologies in electronics has great potential since it allows the integration of sensors, conductors, devices with different electronic functions, etc., in the manufacturing process of three-dimensional objects at an affordable cost, while maintaining freedom in design [4,5].

Among the different 3D printing technologies, the photochemical approach is extremely attractive as objects can be produced via photopolymerization reactions of monomers/oligomers, which possess environmental, economic and production benefits [1]. This approach includes Stereolithography (SLA) and Digital Light Processing (DLP). In DLP, each layer of the resin is exposed all at once to the light source and then cured simultaneously. This allows considerably shorter building times than the point-by-point printing with a laser employed in SLA [2,3,6]. 

Printable materials are mainly composed of mono/multifunctional monomers and/or oligomers, photoinitiating systems and various additives. When the resin is exposed to UV light, the photoinitiators decompose into free radicals that react with the monomer and oligomers, causing them to covalently bond to form long cross-linked polymer chains [1,3,5]. Absorbing additives are commonly used to adjust the vertical resolution, that is, the smallest possible layer thickness [2]. To obtain functional materials as conducting polymer composites suitable for 3D printing, different conductive fillers can be added such as metal nanoparticles, carbon nanotubes, graphene, or intrinsically conducting polymers (ICPs) such as polypyrrole or polyanilines [7,8,9,10]. Polyanilines (PANIs) seem to be excellent candidates for fabricating sensor devices, energy conversion and storage, or supercapacitors due to their intrinsic electrical properties [11,12,13], together with good environmental stability, simple synthesis and low cost [14]. Notwithstanding, its use in 3D printing via vat polymerization is scarce due to challenges such as overlapping UV–Vis absorbance of PANI and the photoinitiator, poor dispersion in the polymer matrix and viscosity at high filler concentrations [3,15]. Only a few recent reports have explored the possibility of incorporating PANI into formulations for DLP printing either individually [16] or combined with graphene [17].

Previous works have studied the influence of the composition of the photocurable resin on the feasibility of DLP printing. Kowsari et al. [18] investigated the effect of different parameters such as photoinitiator, absorber, monomer molecular weight or crosslinker on the resolution and quality of the printed structures. Gojzewski et al. [19] focused on the study of the nanomechanical properties at the interface of polymers printed by DLP using atomic force microscopy (AFM) that showed the effect of light absorbers on the stiffness decay across the individual layers. Hoftstetter et al. [20] studied the curing behavior of mixtures with variable amounts of light absorbers and photoinitiators intended for DLP. They combined photorheology, FTIR and the Jacobs working curves to optimize their content in the formulation, although no 3D printing tests were carried out. The combination of these techniques seems especially interesting for polymers with low curing speed and reduced cross-linking density, where the polymer network and the resulting mechanical properties need time to build up [20]. Jacobs working curves are commonly used to fix the correct settings for DLP [21], as they provide two key parameters of photocurable resins, the penetration depth of the curing light (*D_p_*) and the energy required for the polymerization (*E_c_*) [22]. However, although *E_c_* is an important value, the strength and elastic modulus of a polymer at the gel point are normally too low to ensure successful fabrication during the SLA or DLP process [2]. Thus, the complementary information provided by other techniques as photorheology or photo-DSC to determine gel time [23] can help to understand the printing process. 

The main target of this work is the printability assessment of a conductive, cost-effective, photocurable acrylic resin for DLP. The present paper continues previous research where PANI-HCl was incorporated, as conductive filler, into an in-house resin composed of Ethyleneglycolphenylether acrylate (EGPEA) as the principal monomer, 1, 6-Hexanediol diacrylate (HDODA) as the crosslinker and Diphenyl (2,4,6-tri-methylbenzoyl) phosphine oxide (TPO) serving as the photoinitiator [24].

In the current work, the effect of adding PANI on the printability of the resin was systematically investigated. The concentration of the photoinitiator was also modified to balance the expected slowdown of the photopolymerization reaction due to absorption of PANI at the printing wavelength. The formulations were evaluated by the combination of three techniques, namely, photorheology, Jacobs working curves and DLP printing. From Jacobs working curves, the penetration depth of curing light (*D_p_*) and the energy required for the polymerization (*E_c_*) were calculated to define the best theoretical conditions for printing. 

Further, to relate the printability performance of the novel conducting resins with their microstructure and physico-chemical properties, the printed samples were characterized by Scanning Electron Microscopy (SEM), Attenuated Total Reflectance-Fourier Transformed Infrared Spectroscopy (ATR-FTIR), elemental analysis, electrical conductivity, thermal and mechanical properties both by dynamic mechanical analysis (DMA), differential scanning calorimetry (DSC) and tensile test.

The printing viability of different PANI composites was successfully predicted based on the gel point times determined by Photorheology, plus the Jacobs curve parameters, *E_c_* and *C_d_*. The systematic study revealed that increasing the concentration of TPO allowed overcoming the negative effect of PANI on curing performance to a limited extent. Therefore, for each level of PANI tested, there was an optimal amount of photoinitiators above which no additional benefit was observed. Further, the adjustment of the printing parameters considering *C_d_* enabled printing of most of the tested formulations, except those with the highest gel point times. In short, this work provides valuable guidance for future development of printable cost-effective composites containing conductive UV absorbent fillers. 

## 2. Experimental Section

### 2.1. Materials

Aniline (ANI, 99.5%) and ammonium persulfate (APS, 99%) were obtained from Sigma-Aldrich (St. Louis, MO, USA) and Acros (Geel, Belgium), respectively. Hydrochloric acid (37% HCl) and methanol (MeOH) were purchased from Scharlau (Sentmenat, Spain). Water was purified on an Elix 3 system (Millipore, Molsheim, France). The acrylic monomer and crosslinker employed for polymer synthesis were Ethylene glycol phenyl ether acrylate (EGPEA, molecular weight = 192.21 g mol^−1^) and 1,6-hexanediol diacrylate (HDODA, molecular weight = 226.27 g mol^−1^), respectively. The photoinitiator used was Diphenyl (2,4,6-trimethylbenzoyl) phosphine oxide (TPO, molecular weight = 348.37 g mol^−1^). Monomer, crosslinker and photoinitiator were purchased from Sigma-Aldrich (St. Louis, MO, USA). We employed 2-Propanol obtained from Scharlau (Sentmenat, Spain) to rinse the samples after 3D printing.

**Preparation of PANI.** The PANI used in this paper was synthetized in our laboratory as described in a previous article [24]. The procedure is based on that published by Park et al. [25] and adapted by Horta-Romarís et al. [11]. In brief, the polymerization reaction was performed under mechanic stirring (350 rpm) at room temperature for 3 h. The reaction was initiated by the dropwise addition of the APS oxidant for around 30 min. After 24 h, the resulting product was filtered under vacuum and washed with a mixture of 1:1 water:MeOH. The PANI-HCl was obtained as a dark green powder after drying for 24 h at 80 °C in vacuum in freeze drying equipment (Telstar Lyoquest, Terrassa, Spain). PANI was characterized by UV-Vis spectroscopy, SEM, elemental analysis, FTIR and electrical conductivity as explained in the corresponding sections. 

**Preparation of in-house formulation.** The monomer mixtures were formulated taking as reference a previous study, Arias-Ferreiro et al. [24]. A fixed quantity of the HDODA crosslinker (15 wt%) was added to the principal component, the monomer (EGPEA). Different amounts of conductive filler (PANI 2 wt% to 5 wt%) and photoinitiator (TPO, 4 wt% to 10 wt%) were additionally incorporated. The composite formulations are compiled in Table 1. 

With the aim of promoting homogeneous dispersion, the components were placed into vials and sonicated for 45 min using a Digital Sonifier at 15% intensity (Branson 450). Immediately prior to use, the liquid formulations were further mechanically stirred in a Vortex mixer for 10 min at 1000 rpm (VELP Scientific). After mixing, the formulations were cured in two different ways, depending on the type of analysis and characterization: the first set of samples was cured under the LED lamp and the second set in the 3D printer. 

In order to follow the evolution of the material properties during the photopolymerization reaction, the first set of samples was polymerized under a Visicure 405 nm lamp with an 8 mm diameter focus lens connected to a LED Spot-Curing System (BlueWave, Dymax Corp., Torrington, CT, USA). The UV intensity was adjusted to reach a final intensity of 3 mW cm^−2^ over the sample, measured with a LED radiometer (Dymax ACCU-CAL 50-LED PN40519, Dymax Corp., Torrington, CT, USA). After being subjected to an average exposure time of 999 s to ensure high degrees of conversion, discs of 25 mm in diameter and 200 µm in thickness were obtained.

**Scheme 1:**
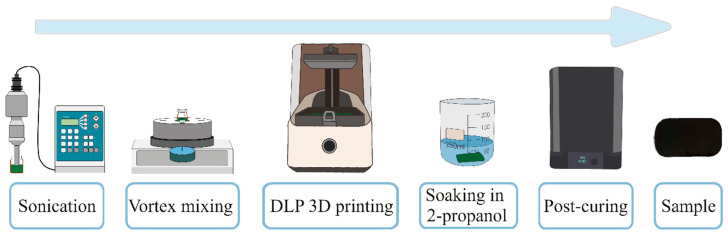
Diagram of sample preparation.

A second set of formulations were cured with a 3D printer (SLASH PLUS, UNIZ, San Diego, CA, USA) which employs LCD Stereolithography printing technology. The printer settings were adjusted based on the printer technical requirements and the composition of the formulations tested. The light irradiation dosage was 76.5 mJ cm^−2^ for each layer, considering that the light intensity of the 3D printer was 3 mW cm^−2^ and the exposure time was 25.5 s per layer. The layer thickness was set at 0.05 mm or 0.025 mm depending on the formulation. After printing, the samples were soaked in 2-propanol for 10 min in order to remove the non-cured resin. A post-curing process was performed with a post-curing lamp (Form Cure, Formlabs, Somerville, MA, USA) for 5 min at 35 °C. To carry out the different tests, flexible rectangular films of 50 mm × 35 mm × 0.5 mm were prepared. The entire preparation process for 3D printing and the aspect of the composite samples is illustrated in Scheme 1. In addition, the formulations were validated by printing objects of different shapes; the digital models were designed and converted to STL file format for 3D printing.

### 2.2. Materials Characterization

The UV-Vis spectra of PANI solution of 60 ppm in EGPEA: 15 wt% HDODA were recorded on a Jasco V-750 double-beam UV-Vis spectrophotometer (Jasco Analítica S.L., Madrid, Spain) between 200 and 800 nm. 

The viscosity of the liquid monomer formulations was determined at room temperature using a controlled strain rheometer (ARES, TA Instrument, Newcastle, DE, USA) with parallel-plate geometry (25 mm diameter, 1 mm gap). The steady shear viscosity (*η*) was measured in a range of shear rates between 0.3 and 100 s^−1^.

**Scheme 2:**
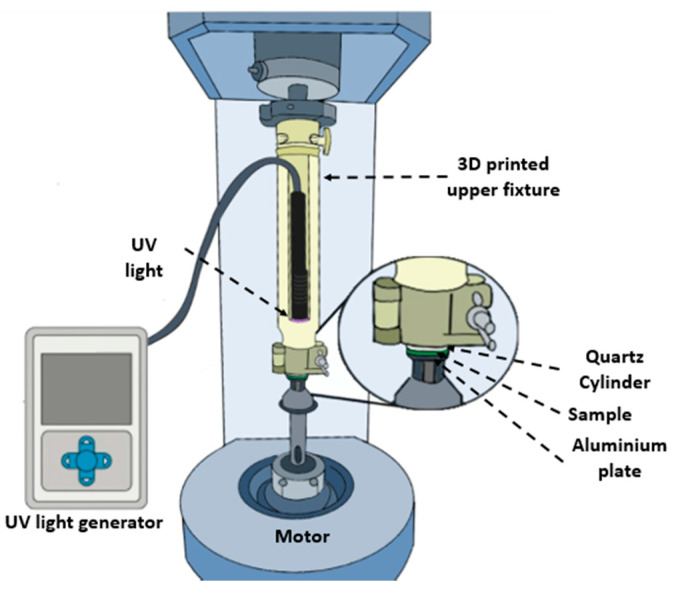
Diagram of the photorheology set-up.

The evolution of the photopolymerization reaction was monitored by in situ plate to plate photorheology in the aforementioned ARES rheometer coupled with a UV lamp. A home-made device, quite similar to that described by Schmidt et al. [26] was employed. The device is depicted in Scheme 2. It consists of a 3D-printed, home-designed upper fixture that allows coupling of the UV source and the upper plate. The upper plate was made of quartz to ensure transmittance of UV irradiation. The liquid sample was placed between the quartz plate and a disposable aluminum parallel plate, both with a diameter of 18 mm [26,27,28].

To follow the evolution of the viscoelastic properties of the composites, a liquid sample was placed between the two plates; then, the light intensity and the gap size between the glass plate and the measuring system were adjusted to approach the conditions of the 3D printer. Due to this, an intensity of 3 mW cm^−2^ was selected and the gap was set at 150 µm. The chosen gap was higher than the size of the PANI agglomerates in the resin suspension. In addition, this is an optimum sample gap for rheological characterization because at a sample thickness <50 µm, the rheological information becomes unreliable, and at a sample thickness of >500 µm, a uniform photocuring reaction cannot be ensured [29].

A time-sweep measurement was carried out for each formulation and the effect of both PANI and TPO concentrations were studied. The tests were performed in dynamic mode with a frequency of 10 rad s^−1^ and a strain of 30%. Prior to this, strain and frequency scans were performed to determine if these conditions lie in the linear viscoelastic range of the materials. The test procedure was divided in two stages. In the first one, the sample was maintained between the plates 300 s without UV light exposure in order to equilibrate it and obtain the baseline for the uncured composite. In the second stage, the photopolymer was cured with a light intensity of 3 mW cm^−2^ for 1300 s [20,30]. During the time-sweep measurements, the evolution of the elastic modulus (*G*′) and loss modulus (*G*″) and tan *δ* were monitored for the composites. At least three measurements at room temperature were made for each formulation.

The Jacobs working curves were calculated for all formulations described in Table 1. To elaborate, the Jacobs working curves the liquid monomer formulations were further prepared following the procedure described in the Section 2.1. Circular films of 18 mm diameter and 1 mm thickness were cured between two glasses under a 405 nm Visicure lamp, with a constant intensity of 3 mW cm^−2^ and varying the exposure times. Every sample was performed in duplicate. After polymerization, the uncured material was removed from the circular films by spraying with 2-Propanol and the remaining solvent was evaporated at room temperature overnight in a fume hood. The thicknesses of the samples were measured with a thickness gauge (Millitast 1080, MAHR GmbH, Göttingen, Germany) and plotted as a function of Exposure (*E_max_*) (mJ cm^−2^).

The Jacobs working curve provides information on two key parameters of photocurable resins: the penetration depth of the curing light and the energy required for polymerization (Equation (1)) [22].

(1) $$C_{d}=D_{p}\cdot \ln\left(\frac{E_{max}}{E_{c}}\right)$$

where *C_d_* (µm) is the cured depth, *E_max_* (mJ cm^−2^) is the light irradiation dosage on the surface, *E_c_* (mJ cm^−2^) is the starting point for the transition from liquid to solid and *D_p_* (µm) represents the penetration depth. This expression equates to a linear line on a semilogarithmic plot of *C_d_* in *y*-axis versus *E_max_* in *x*-axis. The interception of the Jacobs working curve with the *x*-axis represents *E_c_*, whereas *D_p_* is the slope of the linear line. 

The morphology of PANI powder and the printed composite films was evaluated using Scanning Electron Microscopy (SEM). Printed specimens were broken under cryogenic conditions and then examined using a JEOL JSM-7200F Field Emission Scanning Electron Microscope (JEOL Ltd, Tokyo, Japan) at an accelerating voltage of 10 kV, equipped with an Energy Dispersive X-rays Spectrometry System (EDS) for chemical microanalysis (AZtecLive Nanoanalysis, Oxford Instruments, Oxford, UK). Prior to observation, the samples were sputter-coated with a thin palladium/platinum layer (Cressintong 208HR). 

The Fourier Transformed Infrared (FTIR) data were recorded on a Jasco 4700 spectrometer equipment (Jasco Analítica S.L., Madrid, Spain). Three absorption spectra of PANI-HCl were performed in Potassium Bromide (KBr) pellets and the average spectrum examined. The rectangular printed films were analyzed in the Attenuated Reflectance Mode (ATR) by using a MIRacle ZnSe Single Reflection Horizontal ATR accessory (PIKE Technologies, Madison, WI, USA). Three individual spectra were collected on each film. All the spectra were carried out from 4000 to 600 cm^−1^ with a 4 cm^−1^ resolution over 64 scans and subjected to baseline and ATR correction. The spectra were analyzed using the Bruker OPUS^®^ software version 5.5 (Bruker Española S.A, Madrid, Spain). The degree of the acrylate double bonds conversion (DBC%) in the post-cured printed films was calculated from the IR peak area of the band located at 810 cm^−1^, normalized to the carbonyl ester stretching band (*ν*_C=O_) of the acrylic polymer at 1728 cm^−1^, as internal reference, according to Equation (2) [31]:

(2) $$\mathrm{Degree}\;\mathrm{of}\;\mathrm{conversion}\;\left(\mathrm{DBC}\%\right)=\frac{{\left(A_{810}/A_{1728}\right)}_{t=0}-{\left(A_{810}/A_{1728}\right)}_{t}}{{\left(A_{810}/A_{1728}\right)}_{t=0}}\times 100\%$$

The elemental analysis of C, H, N and S of PANI and the composites were conducted in duplicate using ThermoFinnigan FlashEA1112 elemental analyzer (Conquer Scientific, Poway, CA, USA). 

The electrical conductivity (*σ*) at room temperature was calculated from the electrical resistance data by the four-probe method (MCP-T610 LORESTA-GP, Mitsubishi Chemical Corp, Tokyo, Japan) in the samples obtained by 3D printer process. In the case of PANI, the electrical resistance was determined on three square compression molded pellets of 2.5 cm × 2.5 cm × 0.5 mm. The electrical conductivities (*σ*) reported for each polymer formulation are the mean values of at least 12 readings measured on three different samples.

Dynamic mechanical analysis (DMA) was performed on a DMA model Q800 from TA instruments (Newcastle, Delaware, USA). The analysis was used to determine the modulus, glass transition temperature (*T_g_*) and cross-linking density. The cross-linking density, *ν_c_*, is defined as the number of moles of elastically effective network chains per cubic centimeter of film. It was calculated using Equation (3) [31,32,33], where *E’_min_* is the minimum storage modulus and $T_{{E^{\prime }}_{min}}$ is the temperature at minimum storage modulus:

(3) $$\nu_{c}=\frac{{E^{\prime }}_{min}}{3RT_{E^{\prime }min}}$$

The average molecular weight of chain segments between the cross-links, $M_{c}\;$, was calculated for each formulation using Equation (4) [31], where *ρ* is the film density and R is the universal gas constant. *ρ* was determined by the Archimedes principle with a SartoriusLA230S electronic balance equipped with a density measurement kit, using distillate water as the reference liquid.

(4) $$M_{c}=\frac{3\rho RT_{E^{\prime }min}}{{E^{\prime }}_{min}}$$

Rectangular test specimens 15 × 6 × 0.5 mm were prepared for DMA measurements in the 3D printer. The test method was a tension-film mode under the following conditions: frequency of 1 Hz, amplitude of 15 µm and temperature from −40 °C to 80 °C at a heating rate of 4 °C min^−1^.

The glass transition temperatures (*T_g_*) were also evaluated by Differential Scanning calorimetry (DSC) (2010 TA Instruments, Newcastle, Delaware, USA) under nitrogen atmosphere. Then, 10–12 mg of the composites films were put in aluminum pans and heated from −40 °C to 200 °C at a rate of 10 °C min^−1^.

Tensile stress–strain properties were characterized using an Instron 5569 universal testing machine (Instron Canton, Norwood, MA, USA) operating at room temperature and at cross-head speed of 10 mm·min^−1^ until failure. Young modulus (*E*), strength and strain at break point were calculated from stress–strain curves as the average of five dumbbell-shaped specimens of each material of dimensions 30.0 × 3.2 × 1.2 mm.

## 3. Results

### 3.1. Rheological Results

#### 3.1.1. Viscosity of Liquid Formulations

Initially, the effect of both PANI and TPO concentrations on the viscosity of the liquid formulation was studied at room temperature. An adequate viscosity must be tuned for successful 3D-printing. Otherwise, if the viscosity value is too high, the liquid formulation cannot fill the gap between the vat and the previous polymerized layer homogeneously in the specified time, resulting in failure when the building platform is raised during the printing process [7]. 

By way of example, Figure S1 depicts the effect of the filler addition on steady shear viscosity as a function of shear rate for a constant TPO initiator content of 4 wt%, whereas Figure S2 describes the effect of the photoinitiator content for a constant PANI concentration of 5 wt%. Further, the viscosity values obtained at 1 s^−1^ for all formulations assayed are summarized in Table 1. 

A clear augment in viscosity was shown with increasing filler concentration. The presence of PANI perturbed the normal polymer flow; consequently, the viscosity of the filled polymer increased from 0.005 Pa s up to 0.150 Pa s at 1 s^−1^ upon raising PANI loadings from 0 to 5 wt%. These values represented an increase of two orders of magnitude. By contrast, no remarkable effect of the photoinitiator concentration on steady shear viscosity as a function of shear rate was observed. This result can be appreciated in Figure S2, in which the corresponding graphs remain close throughout the tested range.

#### 3.1.2. Photorheology

**Figure 1:**
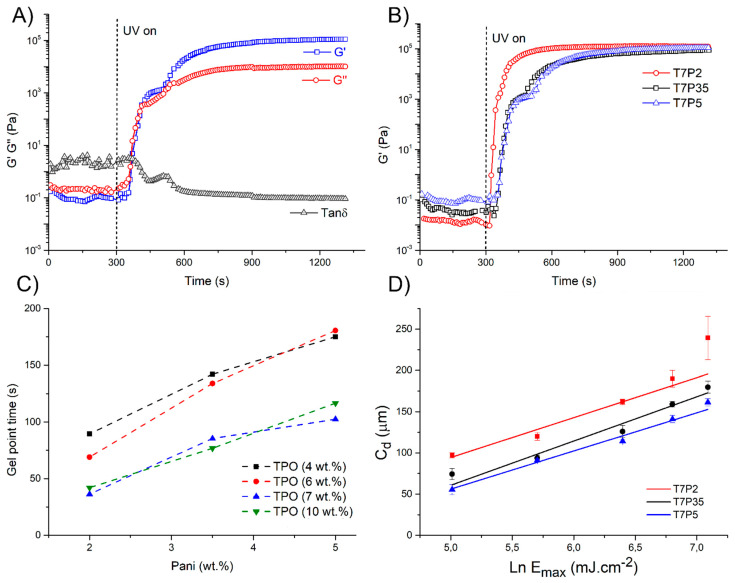
(**A**) Elastic modulus (*G*′), loss modulus (*G*″) and *tan* *δ* versus irradiation time for the composites with 4 wt%. TPO and 5 wt% PANI (T4P5). (**B**) Elastic modulus (*G*′) versus irradiation time for composites with 7 wt% TPO and PANI contents from 2 wt% up to 5 wt% (T7P2, T7P35 and T7P5). (**C**) Gel point time (*s*) versus PANI wt% upon increasing TPO concentration. (**D**) Working curves for composites with 7 wt% TPO and PANI contents from 2 wt% up to 5 wt% exposed to 405 nm light (3 mW cm^−2^).

To further characterize the photopolymerization process, the evolution of the curing reaction was studied in situ. This method monitors the change in modulus during the liquid to solid transition in photocurable composites and can thus determine the gel point. The measured bulk modulus correlates with the degree of polymerization and the crosslinking density [34]. The observed behavior was exactly the same in all photocured composites. As an example, the evolution of the elastic modulus (*G*′), loss modulus (*G*″) and *tan* *δ* in the sample labelled T4P5 is shown in Figure 1A. At the beginning, before turning on the UV lamp and during the first seconds of illumination, *G*′ was smaller than *G*″, corresponding to a liquid-like behavior. Then, the shapes of the *G*′ and *G*″ plots changed over the course of irradiation. After several seconds of exposure time, which was different for each sample, the elastic component increased rapidly; *G*′ and *G*″ curves approached each other, indicating the gel point. From the start of the UV exposure, *tan* *δ* values decreased rapidly due to a restriction in molecular mobility and to the recombination reactions that predominate in great length as the photo-oxidative reaction proceeds. *G*′ increased rapidly, parallel to the decrease in *tan* *δ* exceeding *G*″. This increase was due to the transformation of a viscous Newtonian fluid into an elastic solid during the formation of the network. In the last part of the curve, *G*′ continued to increase slightly indicating the aging process of the gel, while *G*″ remained almost constant. This behavior showed again the dominant elasticity of the system and indicated the formation of a permanent three-dimensional network. 

For clarity, as all composites presented the same features, only the evolution of *G*′ will be shown for samples of increasing PANI content and a selected TPO concentration. Specifically, Figure 1B reports the conversion curves as a function of irradiation time for composites of increasing PANI loading and 7 wt% TPO. It is evident that by increasing the PANI content, the required irradiation time to reach the maximum storage modulus increases; hence, there is an important decrease in the rate of photopolymerization. The same behavior was observed for the remaining amounts of photoinitiator tested (4%, 6% and 10%) (graphs not shown). The photopolymerization parameters were further modified by varying the amount of photoinitiator for a constant PANI wt%. The shapes of the curves obtained upon increasing the TPO content were similar to those of depicted in Figure 1A,B (graphs not shown). 

Hence, to better compare the effect of the addition of both PANI and the photoinitiator, the gel points were determined in the vicinity of the *G*′ and *G*′*’* crossover [29,35,36,37,38,39]. The results are plotted in Figure 1C. First of all, in accordance with Figure 1B, the rate of polymerization clearly decreased at the same rate the gel point time linearly increased, upon augmenting PANI content (R^2^ = 0.92–0.99). Specifically, the gel point time increased 2-fold, 2.6-fold, 2.8-fold and 2.8-fold when the amount of PANI rose from 2% to 5% for amounts of TPO of 4, 6, 7 and 10 wt%, respectively. This is a very well-known phenomenon, mainly due to the strong absorption of light by the PANI filler around 405 nm as described in the absorption spectrum of Figure S3. This is the critical wavelength on which the UV lamps used for photopolymerization have their highest radiation power. As a result, the level of light available for the photoinitiator diminishes [31] and hinders the printing process. 

Following an opposite trend, the photoinitiator concentration has also a great influence on the time needed for the polymerization process. A small concentration of TPO means less reactive species in the solution, responsible for slower initiation and propagation rates [40], so the time for achieving the gel point is higher, and vice versa. Specifically, the effect of increasing the TPO content from 4 up to 10 wt% reduced the gel point time 0.47-fold, 0.54-fold and 0.67-fold for PANI loadings of 2, 3.5 and 5 wt%, respectively. Thus, by increasing the photoinitiator content, it is possible to overcome the competitive absorption effect of PANI and an increase in both acrylic double bond conversion and the rate of photocuring can be achieved [7]. The effect was more evident for the samples with a high amount of PANI. Moreover, as can be observed in Figure 1C, the biggest reduction in the gel point time was found after changing the TPO concentration from 6 to 7 wt% for a given PANI content, whereas a further increase in the photoinitiator content did not provide a clear benefit in terms of curing time (the gel point times for T7P5 and T10P5 were similar, within experimental error). As stated by previous authors, when a critical concentration of photoinitiator is exceeded, competitive chain propagation and/or gel effects, associated with the higher concentration of free radicals, delay the reaction propagation and the gel point [40]. Simultaneously, the effect of crosslinking may be reduced as a result of limited penetration into the material because of rapid crosslinking of the surface layer; therefore, limited polymer crosslinking degree is achieved [41]. In conclusion, given that a fast polymerization is a key parameter in DLP process, the addition of TPO is beneficial as it reduces the exposition times during printing, although there may be a limit for a given formulation above which an increase in TPO does not improve reaction speed.

#### 3.1.3. Cure Depth

The Jacobs working curve is a valuable tool for testing and comparing the feasibility of new photocurable resins for 3D printing. Knowing the values of *E_c_* and *D_p_*, it is possible to determine the required optimum light irradiation dosage, *E_max_*, the cure depth, *C_d_,* and the step height, *z*, for each printed layer. Bearing in mind the properties of the 3D printed objects, it is necessary to obtain good adhesion between layers. To achieve this purpose, the use of layers with sufficient depth is essential, i.e., *C_d_* > *z*, because the stiffness of a polymer at the gel point (or below it) is too low to endure the printing process [19].

In this section, nine Jacobs working curves, corresponding to the different formulations assayed, were created. As an example, Figure 1D shows the working curves of three selected photopolymer formulations with different amounts of conductive filler and a fixed amount of TPO of 7 wt%. The correlation coefficients (R^2^) of the logarithmic regression lines of Jacobs working curves were between 0.92 and 0.99 for all formulations. Small deviations in linearity may be explained by errors in the calculation of the film width and possible changes in the absorptivity and/or refractive indices of the cured and uncured resins with increasing radiation exposure [18,21]. 

Based on Equation (1) and the *E_c_* and *D_p_* parameters previously determined, *C_d_* was calculated for the exposure 76.5 mJ cm^−2^, which corresponded to the product of the light intensity of the printer 3 mW cm^−2^ and an exposure time per layer of 25.5 s. The effect of PANI content and photoinitiator addition on the parameters *D_p_*, *E_c_* and *C_d_* has been studied (Table 1). 

As expected, increasing the amount of PANI from 2 to 5 wt% greatly augmented the critical exposure (*E_c_*) to induce polymerization. This effect was more pronounced at high levels of photoinitiator, with an increase of 3-fold *E_c_* for 10 wt% of TPO. At the same time, increasing the amount of PANI diminished the value of *C_d_*. These results are explained by the well-known ability of PANI to absorb UV radiation and quench radicals [31], since it plays the same role as an UV blocker. The same effect has been described for the addition of lignin [42] and carbon nanotubes [7]. Moreover, the values of *E_c_* obtained in the present paper (21–69 mJ cm^−2^) are higher than the values reported for commercial non-conductive resins by Bennet et al. [21] (<13 mJ cm^−2^), even at the highest level of photoinitiator, which is also explained by the presence of the PANI.

Concerning the effect of the photoinitiator, the highest influence was also observed in the *C_d_* and *E_c_* values. As the amount of photoinitiator increased, the *C_d_* values augmented to a maximum and the *E_c_* values decreased to a minimum, which varied according to the content of PANI (Table 1). A critical amount of photoinitiator that maximized the gel cure depth of a specific formulation had been previously reported by Lee et al. [43]. In the present research, the trends described for the parameters *E_c_* and C_d_ point to an optimal amount of photoinitiator between 6 and 7 wt%, in accordance with the gel point times obtained in the rheological tests.

In general terms, the penetration depth of the light source (*D_p_*) depends on the extinction coefficient and concentration of both initiator and absorber [44]. In the current study, *D_p_* only clearly decreased upon raising PANI content for the minimum amount of photoinitiator employed, 4 wt% TPO, whereas the amount of photoinitiator had hardly influence on *D_p_*. Further, the obtained *D_p_* values for all the formulations (43–71 µm) lie within the low range of commercial resins without conductive fillers tested by Bennet et al. (2017) [21] (53–568 µm).These low values of *D_p_* and the small changes observed could be explained by the high concentrations of photoinitiator and absorber employed. Low *D_p_* values have the advantage to allow most accurate control of the polymerization process and minimal over-cure [44], although with the disadvantage of higher building times [44]. 

In short, following the criterion *C_d_* > *z*, the formulation with 7 wt% TPO and the lowest PANI content (T7P2) seemed the best candidate for satisfactory 3D printability, as it had the highest *C_d_* (62 µm) and the lowest *E_c_* (21 mJ cm^−2^). 

#### 3.1.4. Printer Tests

This section studies the feasibility of the formulations described in Table 1 to be printed in 3D using DLP printing technology. The printer settings were fixed based on the printer technical requirements and the parameters obtained from the Jacobs working curves, where the printing process is expected to succeed if *C_d_ > z*. As stated in Section 2.1, the light irradiation dosage was 76.5 mJ cm^−2^ for each layer, considering that the light intensity of the 3D printer was 3 mW cm^−2^ and the exposure time was 25.5 s per layer, which is the maximum value allowed by the printer. At the start, the layer thickness (*z*) was set at 50 µm for all formulations. Nevertheless, for those that could not be printed under these conditions (*C_d_* < z), further printing with a layer thickness of 25 µm was tested. 

**Figure 2:**
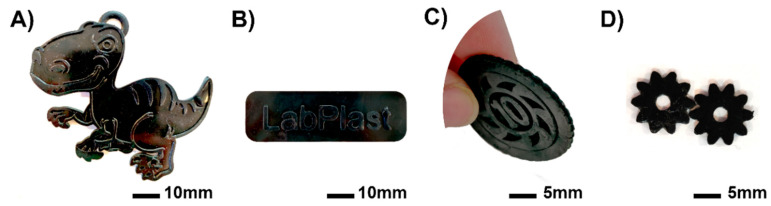
Images of 3D-printed PANI composites with 2 wt% PANI and increasing contents of TPO: (**A**) T6P2; object obtained from Thingiverse, id:2898190 and designed by Tresdlito, (**B**) T6P2, (**C**) T7P2 and (**D**) T10P2.

As summarized in Table 2, the formulations with *C_d_* > 50 µm, corresponding to 2 wt% PANI composites, could be printed in a satisfactory and reproducible way. Further, the formulations with 3.5 wt% PANI, which had *C_d_* values lower than 50 µm (25–45 µm), could still be printed, although with less reproducibility. Finally, considering the *C_d_* values, to be able to print the formulations with the highest PANI content, it was necessary to lower *z* to 25 µm. Nevertheless, the printability of composites with 5 wt% PANI loading and low amounts of TPO (T4P5 and T6P5) could not be fulfilled and only the formulations containing high amounts of TPO could be printed (T7P5, T10P5). These results confirmed that printability is greatly dependent on both the filler content and photoinitiator concentration, and are in agreement with the gel point values depicted in Figure 1C, since to cure formulations with high amounts of PANI and low amounts of TPO, long exposure times were needed. Finally, some printed samples are displayed in Figure 2.

In summary, printability of samples with increasing amounts of PANI can still be achieved by diminishing *z* and increasing photoinitiator to some extent. From there, the long exposure times required for the polymer to build up seem to be the limiting factor that hinders printability. In the present study, this is related to the low power of the printer, insufficient to speed up the process. 

#### 3.1.5. Characterization of PANI and the Printed Films

At the start, the morphology of pristine PANI-HCl was examined by SEM. Similar to previous works, a nanorod morphology was observed. Nanorods of 10–15 nm, made of self-assembled PANI, aggregated in clusters of size ranging from 65 to 95 nm (Figure S4) [24]. Then, the morphological differences between the pure acrylate resin and PANI composites were evaluated at different magnifications. For a constant TPO wt%, upon increasing PANI content micropores appeared and the former smooth surface became rough due to the increasing agglomeration of PANI clusters (Figure S5).

**Figure 3:**
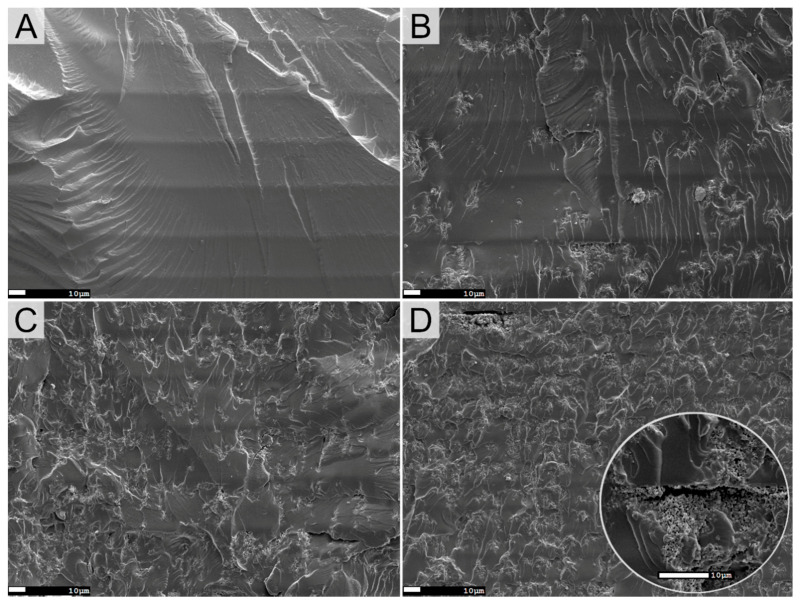
SEM images of (**A**) pure acrylic resin, (**B**) T7P2, (**C**) T7P35 and (**D**) T7P5 with magnitude amplification 500× and inset 1500×.

Similarly, the cryo-fractured cross-section of the film of pure acrylate depicted a flat smooth surface with some features assigned to traces of crack-propagation formed during sample preparation The horizontal lines observed in the images correspond to the individual layers formed during printing and were clearly observed in the pure acrylic resin (Figure 3A). Upon increasing PANI loading, the perception of the layers in the composites is attenuated due to the presence of PANI clusters (Figure 3B–D). This fact is particularly noticeable in Figure 3D, corresponding to T7P5, which has been printed with a *z* value of 25 μm instead of 50 μm. 

Further, the cross-section of the composite films exhibited an increase in the features assigned to crack-propagation and revealed the presence of micropores with clusters of PANI rods protruding from the cavities. For a given TPO content, porosity intensified with PANI loading (Figure 3B–D and D inset), whereas for equal PANI content, lowering the amount of TPO resulted in the appearance of deeper cracks and pores, often located at the interface between consecutive layers. Porosity in vat polymerization techniques may arise from uncured resin between layers. Interlayer porosity may cause weak interfaces and can affect the overall strength of the printed object [45]. These images point to poorer interlayer adhesion upon increasing PANI and lowering the initiator content in agreement with the gel point times and *C_d_* values previously calculated. 

**Figure 4:**
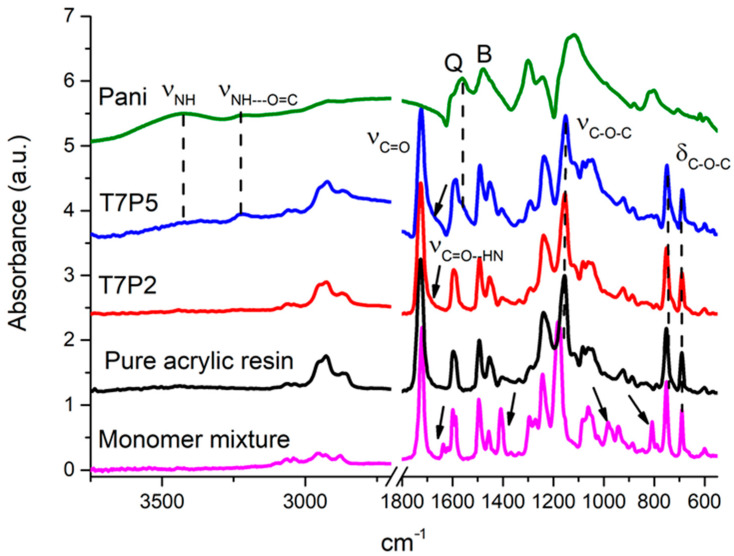
FTIR spectra of monomer mixture, pure acrylic resin, T7P2, T7P5 and PANI.

The effects of PANI and TPO contents on the degree of monomer conversion in the post-cured printed films were studied by ATR-FTIR. As an example, Figure 4 compares the spectrum of the pure acrylic matrix with the spectra of two composite printed films of different PANI content, T7P2 and T7P5 together with the spectra of pure PANI-HCl and the base mixture of liquid monomers. The spectra of the prepolymer mixtures were identical for all the compositions as the suspended PANI particles or the solved TPO could not be detected. 

The spectra of the pristine acrylic matrix and PANI have been thoroughly described in a previous paper [24]. In brief, the main bands that allowed the detection of PANI in the composite spectra were the ones located at 3400 and 3220 cm^−1^, allotted to free and hydrogen-bonded (H-bonded) stretching vibration of PANI secondary amines (ν_NH_), respectively, plus the quinoid (Q, ν_C=N, C=C_) and benzenoid (B, ν_C=C_) bands at 1556 and 1473 cm^−1^, respectively. Regarding the pure acrylic matrix, the strongest bands at 1728 and 1156 cm^−1^ were assigned to the stretching vibrations of the carbonyl bond (ν_C=O_) and asymmetric stretching of the C–O–C bond (ν_C_–_O_–_C_) of the acrylic ester group, respectively. 

In order to assess the degree of the acrylate double bonds conversion (DBC%), the related absorbance bands of the monomer spectrum at around 1636 cm^−1^ (ν_C=C,_ doublet), 1409 cm^−1^ (in plane deformation, scissoring, δ_=CH2_), 984 cm^−1^ and 810 cm^−1^ (out of plane deformation, δ_=CH2_) were compared with the spectra of the cured film [31]. The almost complete disappearance of these bands confirmed very high degrees of monomer conversion in both sides of the film (DBC% = 97.9 ± 0.7 based on Equation (2), Table 3). 

As stated previously [24] and depicted in Figure 4, the ATR profile of 2 wt% PANI composites was clearly dominated by the acrylic matrix bands, whereas upon increasing PANI (≥3.5 wt%), characteristic bands of both filler and acrylic polymer were detected. Parallel to this fact, new bands and peak shifts appeared in the spectra as a result of the H-bond interaction between the carbonyl stretching group (ν_C=O_) of the acrylic resin and the NH groups of PANI (ν_COO_–_HN_). 

The DBC % of printed composites, spanning between 93% and 96%, were slightly lower than the DBC value of the pristine acrylic matrix, whereas the standard deviations were higher (especially composites with high PANI contents). As has been stated in preceding sections, the strong absorption of light by PANI strongly competes with the light adsorption of the photoinitiator [7] and this phenomenon is only partly overcome by increasing the photoinitiator content. Increasing the initiator content above a critical concentration did not necessarily lead to higher conversion; quite the contrary, the DBC % value diminished for the sample labelled T10P5 in relation with the T7P5 formulation, which could be assigned to the small increase in gel time depicted in Figure 1C. This observation indicates that the higher number of disturbed monomers associated with higher TPO contents allows the anticipation of the end of polymerization reaction by the concurrent propagation of the forming chains. This might also result in a lower degree of polymerization and/or crosslinking and a greater amount of residual monomer [40,41]. 

From another point of view, the elemental analysis of PANI lead to 10.92 wt% N; 58.63% C; 5.21% S. The C/N atomic ratio derived from these data, 6.26, is in agreement with the theoretical value for emeraldine doped PANI (C_24_H_20_N_4_Cl_2_) if the counter ion is Cl^−^. The S/N atomic ratio of 0.10 is associated with the use of APS as oxidant [24].

The experimental wt% of PANI in the printed composites was determined utilizing elemental analysis. Since PANI is the only compound in the entire mixture that has the nitrogen in its formulation, the experimental wt% of PANI was calculated using Equation (5):

(5) $$\mathrm{Exp}\;\%\;\mathrm{PANI}=\frac{Exp\;\%N}{4\cdot \frac{At\;wt\;N}{Mw\;\mathrm{PANI}}}\cdot 100$$

Equation (5) is based on the experimental % Nitrogen and the molecular weight of PANI (*M_w_* PANI), assuming that PANI-HCl molecules were completely protonated (50% oxidized poly(p-phenylene amine imine) [46].

Elemental analysis showed that for low amounts of conductive filler (<3.5 wt%), PANI mostly remained in the composite after printing, as the current PANI contents were relatively close to the theoretical amount. As can be seen in Table 3, different yields of the theoretical amount were obtained for each formulation depending on the loading. Specifically, for 2 wt% PANI yields were in the range 77.5−100%, for 3.5 wt% PANI 75.4–87.4% and for 5 wt% PANI, the yields dropped to 60.6–72%. 

According to these data, the maximum amount of PANI that can be incorporated into the acrylic resin during the printing process is around 3 wt%. An increase in viscosity, the tendency of PANI to agglomerate [24] and its interference with the photopolymerization reaction explained these limitations. Possibly, PANI that was not embedded in the resin was removed with 2-propanol during the washing stage after printing. 

From another point of view, PANI-HCl had an average electrical conductivity of 7.25 ± 0.57 S cm^−1^ in the range of good semiconductors; besides, PANI loadings of Table 1 had been selected taking into account the measured percolation threshold of PANI in PEGPA resin, determined in a previous work [24], which was *ρ_c_* = 2.9 ± 0.6 vol% (3.38 ± 0.7 wt%). The electrical conductivity data of the composites of the current work were explained by the elemental analysis outcomes. Despite increasing the amount of conductive charge in the formulation, no significant variations in electrical conductivity were observed for values above 3 wt% (Table 3). Experimental PANI contents below 3 wt% increased electrical conductivity by at least two orders of magnitude with respect to pure acrylic matrix; for PANI ≥ 3 wt%, a maximum increase of three orders of magnitude was obtained. For the current working conditions, the maximum printable concentration of PANI is below the values found by previous authors for PANI-acrylate and PANI-polyurethane compounds, which were 5 wt% and 6 wt%, respectively; nevertheless, the electrical conductivities of the current composites with PANI contents around 3 wt% are five and two orders of magnitude higher, respectively [16,17].

In short, amounts of PANI higher than the limit amount of 3 wt% hindered printability without improving the electrical conductivity, as the additional PANI, unbounded to the polymer matrix was washed away. 

**Figure 5:**
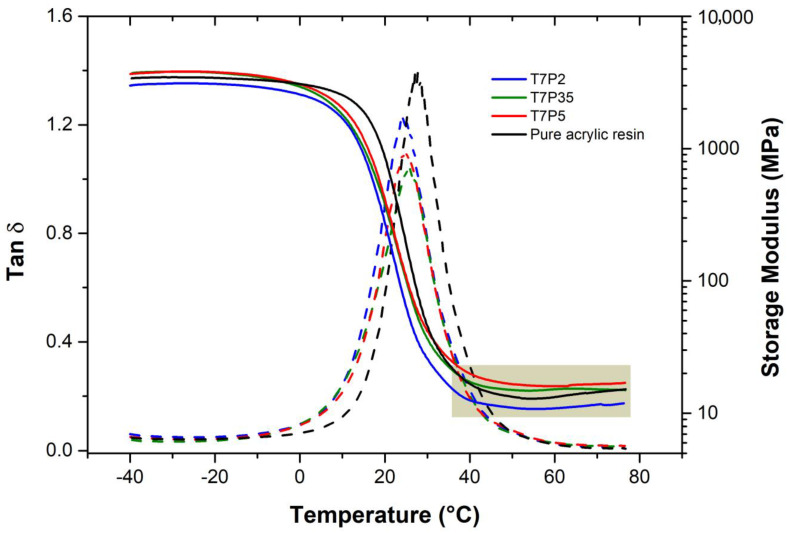
Tan δ (left y-axis and dashed line) and storage modulus (E0) (right y-axis and solid line) for printed films of pure acrylic matrix and composites with 2, 3.5 and 5 wt% PANI content and 7 wt% TPO, obtained by DMA. Maximum in the Tan δ plots represent the *T_g_* of the films measured under the dynamic mechanical test conditions.

Dynamic mechanical analysis (DMA) is a convenient method to study thermal and mechanical viscoelastic properties of polymeric materials and is used to compare the network morphology of the cured films with respect to the average molecular weight of chain segments between the cross-links and the degree of cross-linking [31]. Figure 5 depicts the evolution of *tan* *δ* (left *y*-axis) and storage modulus (*E*′) (right *y*-axis) as a function of temperature with increasing PANI contents prepared at constant 7 wt% TPO. The maximum in the *tan* *δ* plots represent the *T_g_* values under the mechanical test conditions and the *E*′ data are used to calculate the equilibrium rubbery tensile modulus at temperatures far above *T_g_* (*E*′_min_) (the region is highlighted in gray in Figure 5). From the *E’_min_* values and Equations (3) and (4), average *υ_c_* and *M_c_* have been estimated for the tested samples (Table 4). 

As shown in Table 4, the *T_g_* of the pure acrylic resin is 2 °C higher than the corresponding values of the composites; besides, no variation is observed in the *T_g_* values of the composites (around 25 °C) as function of PANI or TPO content, within experimental error. This behavior is not surprising due to the fact that all the formulations have 15% by weight of HDODA crosslinker. The small increase in the *T_g_* of the pure acrylic resin may be related to the higher degree of double bond conversion in the absence of PANI. The same trend is perceived when analyzing the *T_g_* data obtained by DSC, despite the greater experimental error and the fact that the average *T_g_* calculated by this technique (around 13 °C) is lower than that obtained by DMA, proving the rubbery nature of the films (Supplementary Materials, Table S1). 

From another point of view, the cross-link density (*υ_c_*) correlates with the storage modulus in the rubbery state in homogeneous networks [35]. In composite samples or complex systems, the stiffness is the result of a complex interplay between physical and chemical cross-links and the intrinsic rigidly of the filler [47]. Specifically, the *υ_c_* values compiled in Table 4 reflect both the covalent bonds between acrylic chains and the H-bond interactions between the acrylic matrix and PANI filler. As seen in Table 4, the cross-link density (*υ_c_*) slightly increased, whereas the length between the cross-links (*M_c_*) decreased, upon adding PANI to the formulation. Hence, despite the same crosslinker content and minor differences in DBC%, this enhancement is expected owing to the rigidity of PANI clusters dispersed among the cross-linked acrylic matrix [48] and the additional H-bond interaction, described in the FTIR section, which increase the interfacial adhesion between PANI and the acrylic network. 

Concerning the role of the photoinitiator, *υ_c_* and *M_c_* are approximately constant, within experimental error, for a given PANI loading. Further, the higher DBC% and the absence of the rigid filler explain the intermediate values of the pure acrylic resin. As a result, the differences are small, and all the composite films and the pure acrylic resin have similar dynamic mechanical behavior. 

To assess the practical application of printed films, mechanical tensile tests were carried out. From the stress–strain curves depicted in Figure S6, the modulus, stress at break and elongation at break of printed composites were calculated and presented in Table S1 (Supplementary Materials) in comparison with pristine acrylic resin. Regarding the latter, it has similar elongation at break but superior modulus and tensile strength than analogous EGPEA-HDODA copolymers prepared by casting by Borrello et al. [49]. The same authors had observed differences in tensile modules between printed and cast specimens of the same chemical composition, showing the influence of manufacturing conditions in mechanical performance. 

In relation with PANI composites, no significant trends are observed upon varying the PANI loading or the initiator content. The random variations are ascribed to experimental error, except for the decrease in the elongation at break of T7P35 and T7P5. First of all, the subtle differences observed in the storage modulus by DMA as a function of PANI loading cannot be detected in tensile tests. The rigidity of PANI chains could be counterpoised by small variations in DBC% or microstructural defects. Moreover, the lower elongation at break values of TP35 and T7P5 can be ascribed to the observed cracks and pores, mostly located at the interface between layers, due to uncured resin between layers as often observed in vat polymerization, above all in heterogeneous composites [45]. Likewise, no significant differences in both the tensile strength and Young’s modulus were found by Joo and Cho for polyurethane-PANI printed composites with PANI contents up to 6 wt%. The authors suggested that the formation of PANI clusters retards the reinforcing effects on the ultimate strength of the PU/PANI composites [16]. On the contrary, graphene sheets or carbon nanotubes provide better mechanical strength and flexibility as fillers of UV-curable printed resins [7,16,50]. 

## 4. Conclusions

A systematic rheological and Jacobs working curve characterization allowed defining the UV-3D printability window of a series of acrylic conductive resins formulated using increasing loadings of PANI as conductive filler and TPO as photoinitiator.

The photorheology proved to be a valuable technique for measuring the evolution of gel times as a function of PANI and TPO. Due to its high UV absorption, the augment in PANI contents increased gel times and *E_c_* but lowered *C_d_*. These changes were, to some extent, counteracted by the photoinitiator. The optimal amount of TPO that maximized cure depth and minimized critical energy was between 6 and 7 wt%, regardless of PANI loading. Most of the formulations could be printed with previous adjustment of the printer settings considering *C_d_*, except those with the highest gel times. The printing test confirmed the best formulation selected according to its *E_c_* and *C_d_* values, namely T7P2.

The microstructure of composites with high PANI contents and/or low TPO amounts was heterogeneous, and the SEM images depicted pores and cracks, mostly at the layer interface, possibly caused by incomplete curing. These porous patterns can negatively affect interlayer adhesion, in line with the greater difficulties encountered during the printing process and predicted by Jacobs working curve parameters. In addition, ATR-FTIR spectra showed a high degree of curing in all the printed composites, although slightly lower than neat acrylic resin.

The elemental analysis outcomes revealed that the maximum amount of nanofiller embedded within the resin was approximately 3 wt%, which led to an increase in electrical conductivity of three orders of magnitude relative to pure resin (up to σ ≈ 10^−5^ S/cm).

The remaining PANI, unbounded to the polymer matrix, was carried away in the washing step. An increase in viscosity, the tendency of PANI to agglomerate and the slowing down of the polymerization rate upon increasing filler loading explained this fact. PANI loadings above 3.5 wt% hindered printability and promoted microstructure heterogeneity, without improving electrical performance. 

The mechanical and dynamo-mechanical properties of the printed composites mainly reflected the response of the crosslinked acrylic matrix with hardly any difference regardless the amount of PANI or TPO. Minor differences in ductility have been ascribed to the samples’ heterogeneity due to the formation of PANI clusters and its influence on the porosity pattern. As a final point, the small decrease in the *T_g_* compared to the acrylic resin was ascribed to a lower degree of conversion. 

In summary, the developed strategy will provide a practical approach for AM of cost-effective, flexible acrylic nanocomposites with multi-functional properties. Specifically, the conductive composites developed in the current research proved good overall performances, opening new possibilities for application in DLP printing. 

## Figures and Tables

**Table 1:** Composite formulations, penetration depth (*D_p_*), critical exposure (*E*_c_) and cure depth (*C_d_*) calculated from the Jacobs working curves and Viscosity values obtained at 1 s^−1^.

Sample	TPO (wt%)	Theoretical PANI (wt%)	*D_p_* (µm)	*E_c_* (mJ cm^−2^)	*C_d_* (µm)	Viscosity (Pa.s) 1 s^−1^
T4P2	4	2	71	42	43	0.047
T4P35	4	3.5	55	42	34	0.072
T4P5	4	5	59	65	10	0.159
T6P2	6	2	63	34	50	0.051
T6P35	6	3.5	48	30	45	0.059
T6P5	6	5	52	51	21	0.153
T7P2	7	2	48	21	62	0.071
T7P35	7	3.5	54	48	25	0.078
T7P5	7	5	46	44	25	0.155
T10P2	10	2	43	21	56	0.025
T10P35	10	3.5	49	45	26	0.078
T10P5	10	5	49	69	5	0.151

**Table 2:** Printability of the PANI composites (✔ ✔ ✔: very well; ✔ ✔: well; ✔: with difficulties; **x**: not printable).

		TPO (wt%)			
		4	6	7	10
PANI (wt%)	2	✔ ✔	✔ ✔ ✔	✔ ✔ ✔	✔ ✔ ✔
	3.5	✔ ✔	✔ ✔	✔ ✔	✔ ✔
	5	x	x	✔	✔

**Table 3:** Theoretical (T) and experimental amounts of PANI inside the composites by elemental analysis (EA), electrical conductivity of acrylic-PANI composites and double bond conversion (DBC %).

Sample Formulation	PANI (T) (wt%)	PANI (EA) (wt%)	Conductivity (S cm^−1^)	DBC%
Reference	0	<0.05	≤10^−8^ *	97.9 ± 0.7
T4P2	2	2.10 ± 0.11	(3.46 ± 0.50)·10^−6^	96.3 ± 1.4
T4P35	3.5	3.06 ± 0.09	(1.68 ± 0.25)·10^−6^	93.7 ± 1.5
T6P2	2	1.71 ± 0.03	(1.76 ± 0.51)·10^−6^	95.4 ± 0.5
T6P35	3.5	2.86 ± 0.19	(1.39 ± 0.22)·10^−6^	94.9 ± 1.0
T7P2	2	1.60 ± 0.05	(1.59 ± 0.06)·10^−6^	95.7 ± 1.5
T7P35	3.5	3.03 ± 0.05	(2.20 ± 0.37)·10^−5^	94.4 ± 2.2
T7P5	5	3.03 ± 0.01	(1.96 ± 0.96)·10^−5^	95.7 ± 1.5
T10P2	2	1.55 ± 0.07	(3.43 ± 0.56)·10^−6^	96.4 ± 1.5
T10P35	3.5	2.64 ± 0.11	(3.88 ± 0.85)·10^−6^	96.1 ± 1.3
T10P5	5	3.60 ± 0.20	(1.72 ± 0.65)·10^−5^	93.0 ± 2.5

* below the detection limit of the LORESTA-GP device.

**Table 4:** The average molecular weight of chain segments between the cross-links (*M_c_*), cross-link density (*υ_c_*) and transition temperature (*T_g_*) calculated from DMA data for samples obtained by 3D printer.

Sample Formulation	*υ_c_*(·10^−3^) (mol.cm^−3^)	*M_c_* (g·mol)	*T_g_* (°C)
Reference	1.58 ± 0.12	757 ± 59	27.1 ± 0.5
T4P2	1.89 ± 0.21	636 ± 72	25.5 ± 0.6
T4P35	2.00 ± 0.07	589 ± 19	24.4 ± 1.3
T6P2	1.40 ± 0.39	895 ± 248	25.5 ± 0.8
T6P35	1.96 ± 0.18	602 ± 55	25.4 ± 0.1
T7P2	1.75 ± 0.01	687 ± 2	26.1 ± 0.1
T7P35	2.04 ± 0.09	588 ± 26	25.7 ± 0.6
T7P5	2.21 ± 0.01	543 ± 4	24.7 ± 0.7
T10P2	1.34 ± 0.17	910 ± 116	25.0 ± 0.2
T10P35	1.72 ± 0.22	705 ± 92	25.9 ± 0.2
T10P5	2.11 ± 0.10	562 ± 26	25.2 ± 0.2

## Data Availability

The data presented in this study are available on request from the corresponding author.

## Supplementary Materials

The following are available online at https://www.mdpi.com/article/10.3390/polym13132068/s1, Figure S1. Viscosity values as a function of shear rate and PANI content at room temperature for a constant TPO initiator content of 4 wt%. Figure S2. Viscosity values as a function of shear rate and TPO content at room temperature for a constant PANI content of 5 wt%. Figure S3. UV-visible absorption spectrum of PANI-HCl in EGPEA: 15 wt% HDODA at 60 ppm. Figure S4. SEM image showing the nanorod morphology of PANI-HCl with magnitude amplification 3000×. Figure S5. SEM images of the surface of printed films (A) Pure acrylic resin, (B) T7P2, (C) T7P35 and (D) T7P5 with magnitude amplification 1500×. Table S1. Influence of sample formulation on *T_g_*, determined by DSC, and mechanical properties of printed samples measured by tensile test (*E* = Young’s modulus, *σ* = stress at break and *ε* = elongation at break). Figure S6. Stress-strain curve of Pani-acrylic composites.
