# Supplementary material for: Printability Study of a Conductive Polyaniline/Acrylic Formulation for 3D Printing
Source: Polymers (Basel). 2021 Jun 23;13(13):2068. doi: 10.3390/polym13132068 (PMC8272001; doi:10.3390/polym13132068)
Supplement: Supplementary file 1 [file polymers-13-02068-s001.zip › polymers-1253690-supplementary.pdf]

polymers

Supporting information

Printability Study of a Conductive Polyaniline/Acrylic Formulation for 3D Printing

Goretti Arias-Ferreiro [1], Ana Ares-Pernas [1], Aurora Lasagabáster-Latorre [2], Nora Aranburu [3], Gonzalo Guerrica-Echevarria [3], M. Sonia Dopico-García [1] and María-José Abad [1,*]

[1] Grupo de Polímeros, Centro de Investigacións Tecnolóxicas, Universidade da Coruña, Campus de Ferrol, 15471 Ferrol, Spain; goretti.arias@udc.es (G.A.-F.); ana.ares@udc.es (A.A.-P.); s.dopico@udc.es (M.S.D.-G.)
[2] Dpto Química Orgánica I, Facultad de Óptica y Optometría, Universidad Complutense de Madrid, Arcos de Jalón 118, 28037 Madrid, Spain; aurora@ucm.es
[3] POLYMAT and Department of Advanced Polymers and Materials: Physics, Chemistry and Technology, Faculty of Chemistry, University of the Basque Country UPV/EHU, 20018 Donostia-San Sebastián, Spain; nora.aramburu@ehu.eus (N.A.); gonzalo.gerrika@ehu.eus (G.G.-E.)
* Correspondence: mjabad@udc.es

S1. Results

S1.1. Rheology

Viscosities of the liquid formulations are shown in Figure S1 and Figure S2.

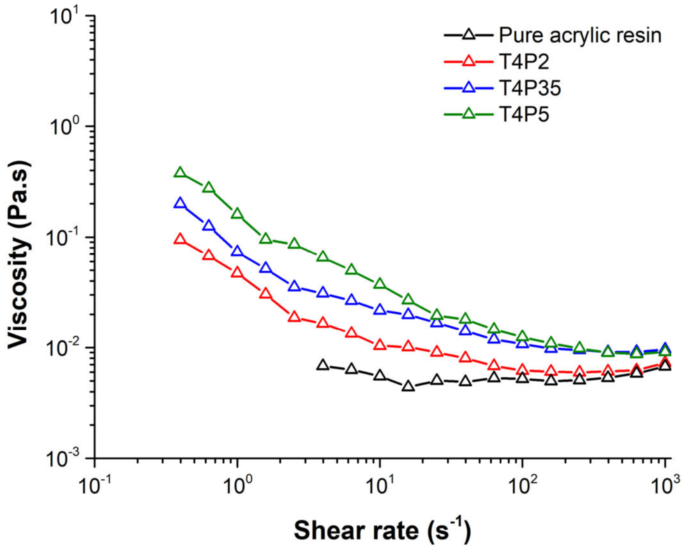

Figure S1. Viscosity values as a function of shear rate and PANI content at room temperature for a constant TPO initiator content of 4 wt%.

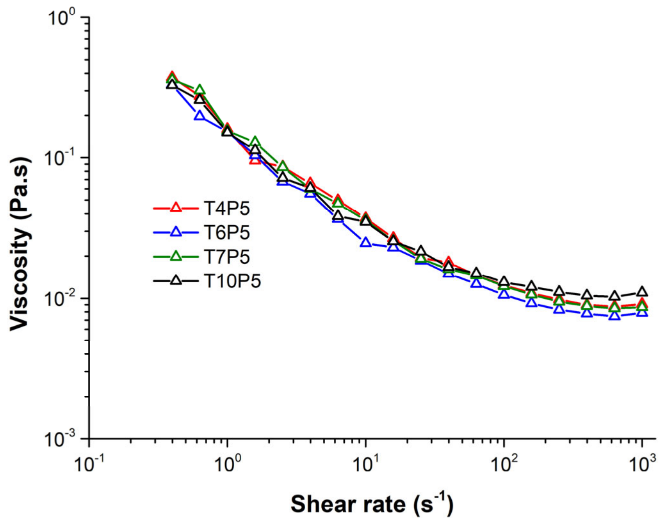

Figure S2. Viscosity values as a function of shear rate and TPO content at room temperature for a constant PANI content of 5 wt%.

S1.2. UV-Visible Spectra of PANI-HCl

Figure S3 shows that the UV-Vis an absorbance spectrum of PANI-HCl dispersed in EGPEA:15 wt% HDODA at a concentration of 60 ppm. The characteristic peaks of emeraldine salt form of PANI are detected at 315, 386, 485 (shoulder but distinctive peak in the first derivative spectrum) and >800 nm. The peak at 386 nm can be assigned to π–π^* transition of the benzenoid, whereas the peaks at around 485 and above 800 nm can be attributed to the polaron-π^* and π-polaron transitions, respectively [1]. The strong absorption around 405 nm (the UV lamp of the 3D printer is a 405 nm model) indicate that PANI will affect the curing process of the acrylate dispersions.

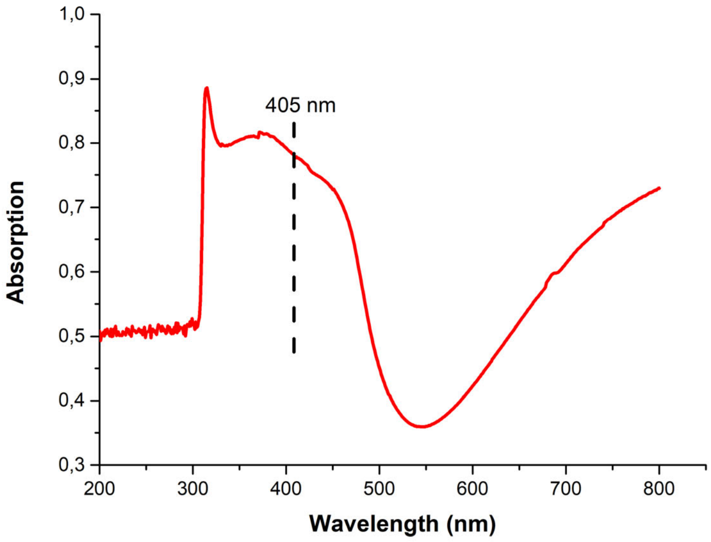

Figure S3. UV-visible absorption spectrum of PANI-HCl in EGPEA:15 wt% HDODA at 60 ppm.

S1.3. Structural Characterization

The smooth surface of the pure acrylic resin and in-house PANI printed composites and the nanorod morphology of pristine PANI-HCl and are shown in Fig. S4 and Fig S5, respectively.

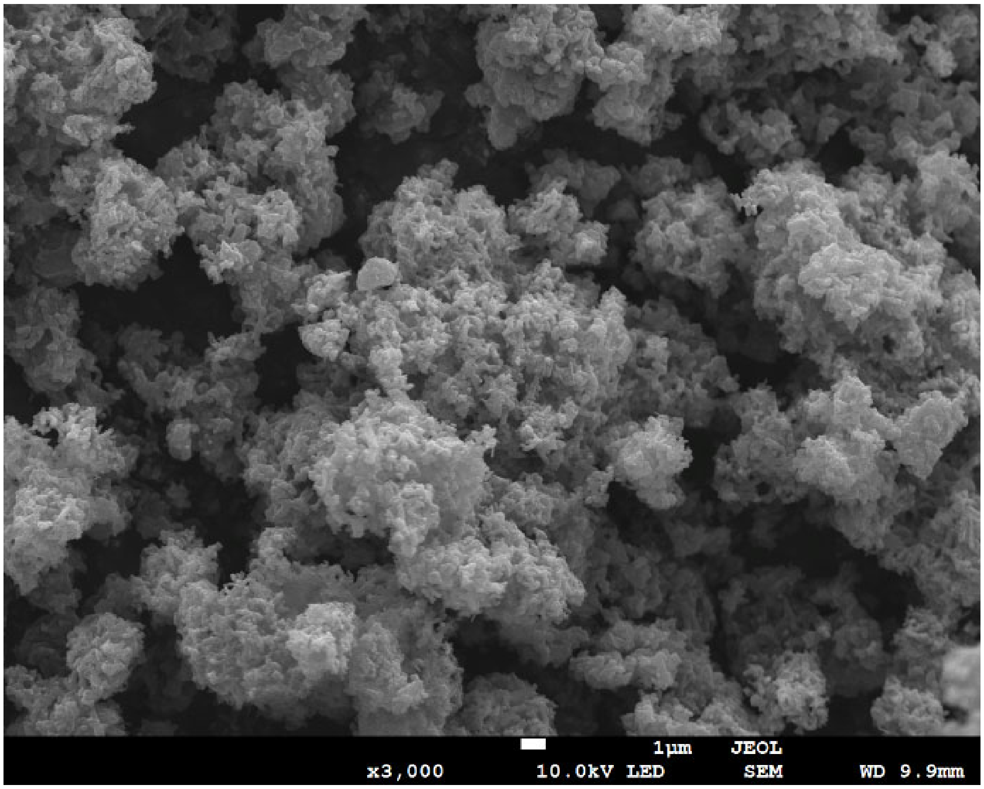

Figure S4. SEM image showing the nanorod morphology of PANI-HCl with magnitude amplification 3000×.

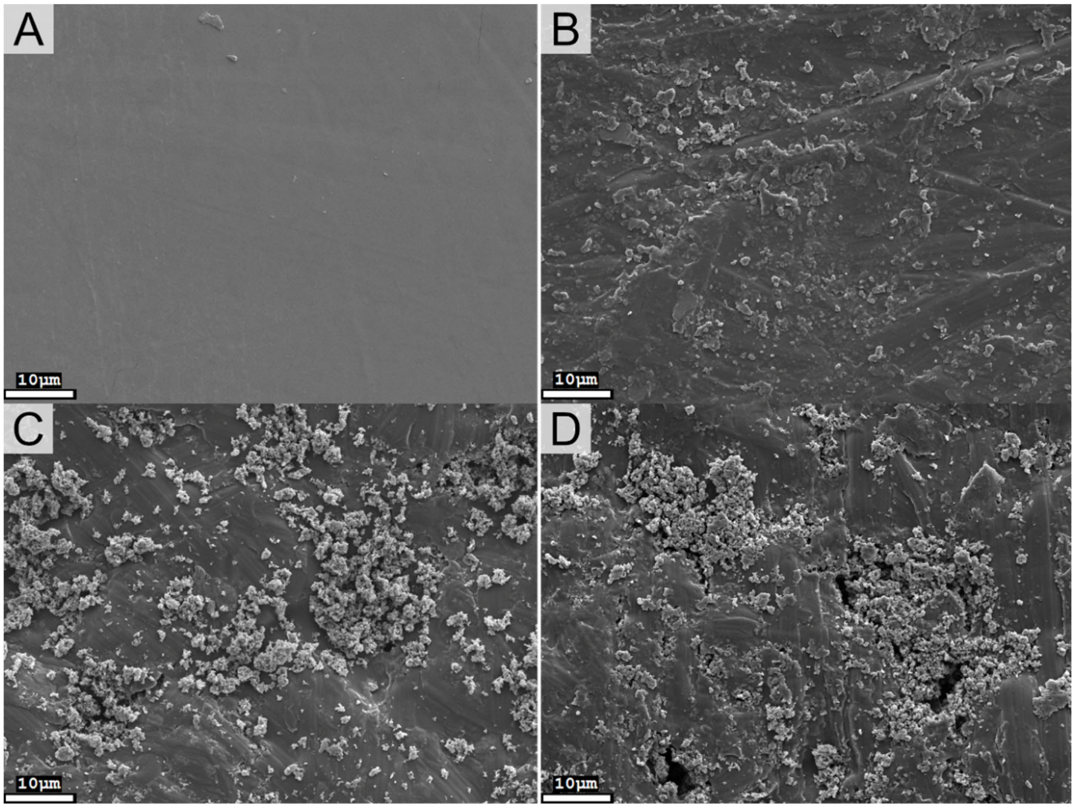

Figure S5. SEM images of the surface of printed films (**A**) Pure acrylic resin, (**B**) T7P2, (**C**) T7P35 and (**D**) T7P5 with magnitude amplification 1500×.

S1.4. Thermal and Mechanical Properties

Table S1. Influence of sample formulation on T_g, determined by DSC, and mechanical properties of printed samples measured by tensile test (E = Young's modulus, σ = stress at break and ε = elongation at break).

**Sample Formulation**	T_g **(ºC)**	E **(MPa)**	σ **(MPa)**	ε **(%)**
**Reference**	16.1 ± 1.5	20.1 ± 1.6	3.5 ± 1.6	20.1 ± 1.6
**T4P2**	13.4 ± 1.8	18.8 ± 1.1	3.0 ± 0.3	17.2 ± 1.2
**T4P35**	10.9 ± 0.5	21.0 ± 4.4	3.8 ± 1.5	18.4 ± 4.9
**T6P2**	13.4 ± 1.3	18.5 ± 1.1	3.1 ± 0.4	18.3 ± 1.9
**T6P35**	9.6 ± 1.1	21.8 ± 0.4	4.2 ± 0.4	20.3 ± 2.3
**T7P2**	14.0 ± 0.0	18.1 ± 1.6	3.0 ± 0.3	18.0 ± 1.2
**T7P35**	12.7 ± 0.1	18.9 ± 1.8	2.3 ± 0.6	12.2 ± 2.1
**T7P5**	12.8 ± 1.2	21.4 ± 1.5	3.3 ± 0.5	15.9 ± 1.8
**T10P2**	15.1 ± 1.1	19.7 ± 1.0	3.5 ± 0.3	20.0 ± 0.8
**T10P35**	14.2 ± 1.0	22.3 ± 2.0	3.9 ± 0.2	19.5 ± 0.5
**T10P5**	14.0 ± 0.3	22.0 ± 2.0	3.7 ± 0.7	17.3 ± 2.0

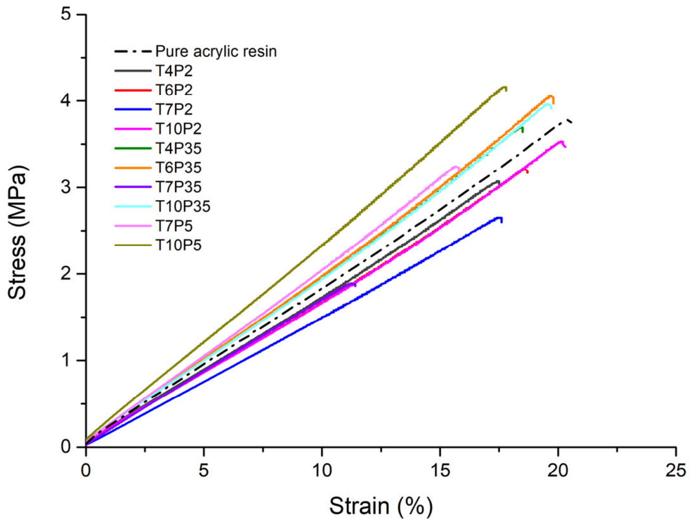

Figure S6. Stress-strain curve of Pani-acrylic composites.

References

1. Jafarzadeh, S.; Thormann, E.; RönnevallT.; Adhikari, A.; Sundell, P.-E.; Pan, J.; Claesson, P.M. Toward Homogeneous Nanostructured Polyaniline/Resin Blends. *ACS Appl. Mater. Interfaces* **2011**, *3*, 1681–1691, doi:10.1021/am2002179.
